# Potential Molecular Targets of the Broad-Range Antimicrobial Peptide Tyrothricin in the Apicomplexan Parasite *Toxoplasma gondii*

**DOI:** 10.3390/biomedicines14010172

**Published:** 2026-01-13

**Authors:** Yosra Amdouni, Ghalia Boubaker, Joachim Müller, Maria Cristina Ferreira de Sousa, Kai Pascal Alexander Hänggeli, Anne-Christine Uldry, Sophie Braga-Lagache, Manfred Heller, Andrew Hemphill

**Affiliations:** 1 Institute of Parasitology, Department of Infectious Diseases and Pathobiology, Vetsuisse Faculty, University of Bern, Länggass-Strasse 122, 3012 Bern, Switzerland; joachim.mueller@unibe.ch (J.M.); maria.ferreira@unibe.ch (M.C.F.d.S.); kai.haenggeli@unibe.ch (K.P.A.H.); 2 Institute of Metrology METAS, Lindenweg 50, Wabern, 3003 Bern, Switzerland; ghalia.boubaker@metas.ch; 3 Proteomics and Mass Spectrometry Core Facility, Department for BioMedical Research (DBMR), University of Bern, Murtenstrasse 28, 3008 Bern, Switzerland; anne-christine.uldry@unibe.ch (A.-C.U.); sophie.braga-lagache@unibe.ch (S.B.-L.); manfred.heller@dbmr.unibe.ch (M.H.)

**Keywords:** *Toxoplasma*, human fibroblasts, antimicrobial peptide, electron microscopy, differential affinity chromatography, proteomics

## Abstract

**Background:** The apicomplexan parasite *Toxoplasma gondii* causes serious diseases in animals and humans. The in vitro efficacy of the antimicrobial peptide mixture tyrothricin, composed of tyrocidines and gramicidins, against *T. gondii* tachyzoites was investigated. **Methods:** Effects against *T. gondii* were determined by monitoring inhibition of tachyzoite proliferation and electron microscopy, host cell and splenocyte toxicity was measured by Alamar blue assay, and early embryo toxicity was assessed using zebrafish embryos. Differential affinity chromatography coupled to mass spectrometry and proteomics (DAC-MS-proteomics) was employed to identify potential molecular targets in *T. gondii* cell-free extracts. **Results:** Tyrothricin inhibited *T. gondii* proliferation at IC_50_s < 100 nM, with tyrocidine A being the active and gramicidin A the inactive component. Tyrothricin also impaired fibroblast, T cell and zebrafish embryo viability at 1 µM. Electron microscopy carried out after 6 h of treatment revealed cytoplasmic vacuolization and structural alterations in the parasite mitochondrion, but these changes appeared only transiently, and tachyzoites recovered after 96 h. Tyrothricin also induced a reduction in the mitochondrial membrane potential. DAC-MS-proteomics identified 521 proteins binding only to tyrocidine A. No specific binding to gramicidin A was noted, and four proteins were common to both peptides. Among the proteins binding specifically to tyrocidine A were several SRS surface antigens and secretory proteins, mitochondrial inner and outer membrane proteins associated with the electron transfer chain and porin, and several calcium-binding proteins putatively involved in signaling. **Discussion:** These results suggest that tyrocidine A potentially affected multiple pathways important for parasite survival and development.

## 1. Introduction

*Toxoplasma gondii* (Apicomplexa: Coccidia) is a tissue cyst-forming protozoan parasite with a worldwide distribution [1,2,3]. *T. gondii* causes the disease toxoplasmosis in animals and humans, and is one of the most successful parasites due to its capacity to infect any mammalian or avian species and its high zoonotic potential [4]. In immunocompetent hosts, infection with *T. gondii* is typically asymptomatic or presents with only mild symptoms. However, the consequences can be far more serious for certain high-risk groups, particularly during pregnancy and in immunocompromised individuals. Upon primary infection during pregnancy, there is the risk of transplacental transmission of the parasite to the fetus, leading to congenital toxoplasmosis. The severity of fetal infection often depends on the timing of maternal infection during gestation, with early infections more prone to result in miscarriage or severe developmental damage, while infections later in pregnancy are more likely to be subclinical at birth but can still cause long-term complications. Clinical symptoms, including ocular complications, can emerge months or even years after infection [5,6] and can cause impaired vision, and at a later stage, blindness [7]. In immunocompromised individuals, recrudescence of *T. gondii* bradyzoites encapsulated in tissue cysts poses a major risk [1]. This reactivation can result in severe toxoplasmosis, which may be life-threatening [4]. The most commonly prescribed treatment for toxoplasmosis involves a combination of sulfadiazine and pyrimethamine, blocking enzymes essential for folate synthesis [8,9]. Alternative therapies include combinations of pyrimethamine and clindamycin or clarithromycin, or applying quinolones such as atovaquone. However, these treatments are not universally effective, may cause significant side effects, and do not eradicate the tissue cysts that sustain chronic infection [10,11].

In the search for new anti-parasitic agents, antimicrobial peptides (AMPs) have attracted attention as a potentially promising class of molecules. These small, naturally occurring peptides are integral components of innate immune responses across virtually all living organisms and are characterized by broad-spectrum activity against bacteria, viruses, fungi, and parasites [12]. AMPs have been found in a wide variety of plant and animal species as well as humans [13]. Furthermore, many peptides produced by different microorganisms have been identified and investigated for their therapeutic potential [14]. Due to their structural flexibility, AMPs represent interesting candidates as therapeutic agents.

Recent studies have shown that several AMPs exhibit potent activity against *T. gondii*. For example, lycosin-I, a peptide derived from spider venom and respective lipopeptide derivatives, was shown to inhibit *T. gondii* tachyzoite invasion and proliferation in vitro and significantly increased survival rates in acutely infected mice [15,16]. Similarly, XYP1, a peptide from wasp venom and its derivatives, displayed antiparasitic effects in vitro by disrupting parasite membranes and impairing mitochondrial function, which increased the life span of acutely *T. gondii*-infected mice by a few days. In addition, a peptide derived from the broad-spectrum AMP leucinostatin has been shown to exhibit profound in vitro activity not only against *Trypanosoma brucei*, *T. cruzi*, *Leishmania donovani*, and *Plasmodium falciparum*, but also against *T. gondii* [17,18]. However, an inherent problem with many AMPs is the broad-spectrum toxicity not only for pathogens, but also for mammalian cells, which renders them mostly suitable for topical applications, but not necessarily for systemic treatments [19].

In this study, the effects of seven AMPs were assessed in vitro using *T. gondii* tachyzoites grown in human foreskin fibroblast (HFF) host cells. These peptides were selected based on prior evidence of their inhibitory effects against other intracellular protozoan parasites such as *Trypanosoma* [20,21,22], *Plasmodium* [23,24], and *Leishmania* [25,26]. One of these AMPs, tyrothricin, is an antibiotic peptide complex produced and extracted from the aerobic Gram-positive bacterium *Brevibacillus parabrevis*. Tyrothricin is a mixture composed of 60% tyrocidine cationic cyclic decapeptides and 40% neutral linear gramicidins with valine-gramicidin A as the major component. We demonstrate the anti-*Toxoplasma* and cytotoxic activities of tyrothricin, show that the activity is predominantly based on its tyrocidine A component, and employ affinity chromatography combined with mass spectrometry-based proteomics (DAC-MS-proteomics) using tyrocidine A and gramicidin A coupled to NHS-activated sepharose to identify potential mechanisms of action affecting multiple pathways in *T. gondii* tachyzoites.

## 2. Materials and Methods

### 2.1. Cell Culture Equipment and Media, Biochemicals and Compounds

Cell culture devices were purchased from SARSTEDT (Sevelen, Switzerland), and culture media were obtained from Gibco-BRL (Zürich, Switzerland). The AMPs used in this study (see Supplementary Table S1 [22,25,27]) were procured from Sigma-Aldrich (St. Louis, MO, USA), except tyrocidine A that was purchased from TOKU-E company (Bellingham, WA, USA). AMPs were received as powders, and stock solutions (10 mM) were prepared in dimethyl-sulfoxide (DMSO) and stored at −20 °C.

### 2.2. Toxoplasma gondii Strains and Human Foreskin Fibroblast (HFF) Host Cells

HFF cells (PCS-201-010TM) were purchased from ATCC (American Tissue Culture Collection, Manassas, VA, USA) and cultured as described by Ramseier [28]. *T. gondii* RH tachyzoites expressing *E. coli* β-galactosidase (*T. gondii*-β-gal, Type I) were obtained from Prof. David Sibley, Washington University, St. Louis, USA, and were used to quantify the anti-proliferative activity of the tested AMPs as previously described [29,30]. *T. gondii* Me49 tachyzoites (Type II) were a kind gift from Dr. Furio Spano, ISS, Rome, Italy, and were also cultured in HFF [31].

### 2.3. Measurement of Anti-Proliferative Activities of AMPs Against T. gondii-β-Gal Tachyzoites

The primary screening of the different AMPs (see Supplementary Table S1) against *T. gondii*-β-gal grown in HFF monolayers was performed by growing HFF to ~80% confluency in T-25 culture flasks and adding the compounds concomitantly to infection at either 0.1 or 1 µM, followed by further culture at 37 °C/5% CO_2_ for 72 h. Then, measurements of β-galactosidase activity were carried out as previously described [32]. For tyrothricin, IC_50_ values (=half-maximal inhibitory concentration resulting in 50% inhibition of proliferation) based on the β-galactosidase activity measurements were determined. For this, serial dilutions (ratio 1:2) of tyrothricin were prepared in culture medium and added either 5 min prior to infection of host cells or 3 h after infection, resulting in pre- or post-infection IC_50_ values. IC_50_ values were calculated by performing regression analysis on a dose–response curve by using Excel software (Microsoft, Redmond, WA, USA) as previously described [33].

### 2.4. In Vitro Assessment of AMP Cytotoxicity Against HFF

The impact of the seven AMPs on HFF viability was determined by resazurin reduction assay [34]. Briefly, HFF monolayers were seeded in 96-well plates at a density of 5 × 10^3^ cells/well until reaching ~80% confluency. The AMPs were added to monolayers at concentrations of 0.1 or 1 μM, and HFF were further cultured at 37 °C/5% CO_2_. After 72 h, the medium was discarded, and viability of HFF was determined by Alamar blue assay as previously described [34].

### 2.5. Assessment of the Effects of Tyrothricin on Proliferation of Murine Splenic B- and T-Cell Populations

The effects of tyrothricin in vitro treatments on the viability of murine B and T cells were performed as described previously [35]. In brief, spleens were aseptically removed from naïve female BALB/c mice, then single-cell suspensions were prepared and seeded in 96-well plates. Splenic lymphocytes were used either unstimulated (negative control) or were stimulated with Concanavalin A (ConA, 5 µg/mL, induction of T cell proliferation), lipopolysaccharide (LPS, 10 µg/mL, induction of B cell proliferation), and ConA plus tyrothricin or LPS plus tyrothricin. Tyrothricin was added at concentration of 0.1, 0.5, 1, or 2 µM. The immunosuppressive agent cyclosporine A (CsA) was included as a control. To assess viability of proliferating T and B cells under different stimulation/treatment conditions, we used Alamar blue assay as described [35,36]. Data comparisons between groups were performed using a Student’s *t*-test using the Microsoft Excel software package (Microsoft, Redmond, WA, USA) [33].

### 2.6. Assessment of the Effects of Tyrothricin on Early Zebrafish Embryo Development

Assays were carried out as previously reported [34,37]. Freshly fertilized zebrafish eggs were kindly provided by the group of Nadia Mercader, from the Institute of Anatomy, University of Bern, and transferred into a Petri dish (Sarstedt Inc., Nümbrecht, Germany) containing E3 medium/osmosis water. Viable eggs were placed individually into 24-well plates containing 1 mL/well of E3 medium/osmosis water plus tyrothricin at 0.2, 1, 10, or 20 µM, with 20 wells per test concentration and 4 internal controls. Additional controls (20 wells each) included negative control (E3/osmosis water), solvent control (E3/osmosis water plus 0.01% DMSO), and a positive control drug (E3/osmosis water plus 50 µM BKI-1750) [34]. Plates were sealed with parafilm and maintained at 28 °C, with solutions replaced every 24 h. Embryo viability and malformations were assessed in a blinded manner under a Nikon Eclipse TS100 microscope (10×) at 24, 48, 72, and 96 h post fertilization (hpf), with plates kept at 26 °C during observation. At 96 hpf, embryos were euthanized with pre-cooled MS222 (100 µg/L; Argent Chemical Laboratories, Redmond, WA, USA) and stored at –20 °C for 24 h. An impact score for each tyrothricin concentration was calculated relative to water and solvent controls as described [34].

### 2.7. Transmission Electron Microscopy (TEM)

HFF monolayers were grown to semi-confluence in T25 culture flasks and were infected with 10^7^ *T. gondii* Me49 tachyzoites for 3 h at 37 °C/5% CO_2_. Subsequently, cultures were exposed to continuous treatments with 0.5 µM of tyrothricin for 6, 9, 12, 24, 48, 72, or 96 h, with a change of fresh medium plus compounds after 48 h, all at 37 °C/5% CO_2_, whereas negative control cultures received culture medium containing 0.005% DMSO (solvent control). The cells were then gently removed from the flasks using a cell scraper. They were washed in 100 mM sodium cacodylate buffer pH 7.3, fixed in glutaraldehyde and osmium tetroxide, and dehydrated in ethanol (30, 50, 70, 90, and 3 × 100%) as previously described [31]. They were finally embedded in Epon-182 epoxy resin, and polymerization of the resin was carried out at 60 ◦C. Ultrathin sections (80 nm) were cut using an ultramicrotome (Reichert and Jung, Vienna, Austria) and placed onto 200 mesh formvar-carbon-coated nickel grids (Plano GmbH, Marburg, Germany). Contrasting was performed with Uranyless^®^ and lead citrate (both from Electron Microscopy Sciences, Hatfield, PA, USA). Samples were inspected on a Philips-FEI Morgagni TEM (Hillsboro, OR, USA) equipped with a Morada digital camera system (12 Megapixel) operating at 80 kV (Media System Lab, Macherio, Italy). Ultrastructural alterations in the parasitophorous vacuoles and in individual tachyzoites (mitochondrial changes, vacuolization, and other cytoplasmic alterations) were assessed by visual inspection of at least 100 parasitophorous vacuoles per specimen.

### 2.8. Tetramethylrhodamine Ethyl Ester (TMRE) Uptake Assay for Assessment of Changes in the Mitochondrial Membrane Potential (MMP)

TMRE uptake assays were performed as described [35,36]. HFF cultures grown in T25 flasks to ~80% confluency were infected with 2 × 10^5^ *T. gondii* ME49 tachyzoites and maintained for 48 h. Cultures were then treated with 0.5 µM tyrothricin, or 0.5 µM pyrimethamine (PYR; MMP—negative control), or 0.005% DMSO (untreated control) for 3 h, or with 80 µM Carbonylcyanid-4-(trifluormethoxy)phenylhydrazon (FCCP; MMP—positive control) for 10 min. Cells were washed with Hank’s balanced salt solution (HBSS), incubated with 500 nM TMRE for 30 min, harvested, passed through a G25 needle, and filtered. Lysates (100 µL/well) were measured for fluorescence (exc. 544/20 nm, em. 590/20 nm) using a Hidex Sense microplate reader (Hidex, Mainz, Germany). Mean TMRE uptake was calculated from three biological replicates, and statistical significance was assessed by Student’s *t*-test (*p* ≤ 0.05) (Microsoft, Redmond, WA, USA) [35].

### 2.9. Differential Affinity Chromatography (DAC) of T. gondii Cell-Free Extract Using Tyrocidine A and Gramicidin Coupled to Sepharose 4 FAST FLOW Matrix

Frozen pellets of *T. gondii* ME49 tachyzoites were resuspended in ice-cold extraction buffer containing 1% Triton X-100 and 1% HALT proteinase inhibitor cocktail (ThermoFisher Scientific, Reinach, Switzerland), vortexed thoroughly, and centrifuged at 10,000× *g* for 30 min. The resulting supernatants were subjected to DAC essentially as described [18].

### 2.10. Proteomics Analyses

The lyophilized eluates were dissolved in 10 μL urea buffer and processed by mass spectrometry exactly as published earlier [38]. The mass spectrometry data were interpreted with Fragpipe version 22.0, leaving out deamidation as a variable modification as the only difference from the earlier work.

## 3. Results

### 3.1. In Vitro Screening of AMPs Against T. gondii and HFF Host Cells and Identification of Tyrothricin

**Figure 1:**
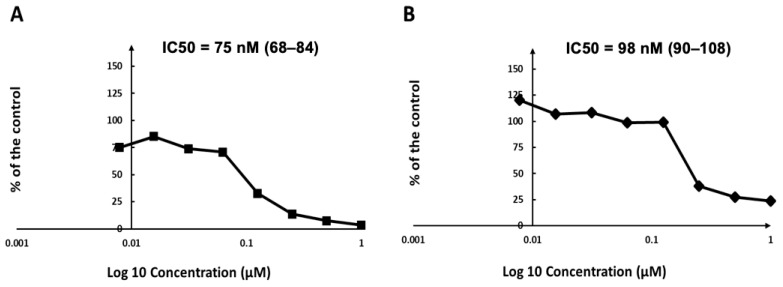
In vitro dose–response curves and *T. gondii* IC_50_ values of tyrothricin applied either concomitantly during infection (**A**) or 3 h post-infection (**B**). Serial dilutions of tyrothricin (ratio 1:2) ranging from 1 to 0.0078 µM were prepared in culture medium and added either 5 min prior to infection of host cells or 3 h after infection. Graphs are presented in % proliferation in relation to non-treated *T. gondii* tachyzoites.

Results from the primary in vitro screening of seven AMPs against *T. gondii* β-gal tachyzoites grown in HFF and against uninfected HFF are summarized in Table 1. Six of the seven AMPs did not impair the proliferation of *T. gondii* tachyzoites, and had no impact on the viability of uninfected HFF when applied at 1 µM. In contrast, tyrothricin inhibited tachyzoite proliferation by 50% at 0.1 μM, and almost completely at 1 µM (Table 1). While HFF viability was only marginally affected at 0.1 µM, tyrothricin treatment at 1 µM resulted in an almost 40% loss of HFF viability. Tyrothricin was further evaluated by dose–response experiments. Addition of tyrothricin concomitantly to infection resulted in an IC_50_ value of 75 nM [68–84]. However, when the compound was added to already intracellular parasites, the IC_50_ value was 98 nM [90–108]. Respective dose–response curves are found in Figure 1.

### 3.2. Tyrothricin Treatment Is Detrimental to the Viability of Murine T Cells and Zebrafish (Danio Rerio) Embryos In Vitro

**Figure 2:**
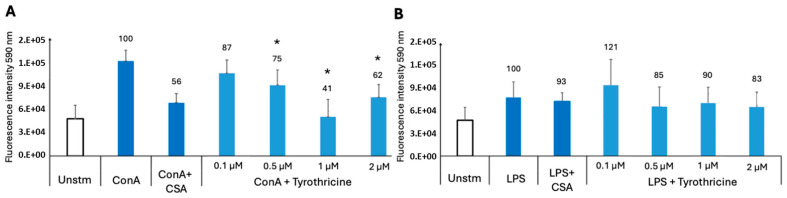
In vitro effects of tyrothricin on viability of stimulated murine T (**A**) and B cells (**B**) by Alamar blue assay. Single spleen cell suspensions were left unstimulated (Unstm) or exposed to ConA (5 μg/mL) or LPS (10 μg/mL). Splenocytes were stimulated with ConA or LPS and concomitantly treated with cyclosporin A (CsA), a known inhibitor of T-cell stimulation, or tyrothricin was added at 0.1, 0.5, 1, or 2 μM to ConA or LPS stimulated cells. A viability level of 100% was set for ConA and LPS controls; * indicates *p* < 0.05 as assessed by Student’s *t*-test.

Murine splenocytes represent a mixed population of different immune cells. To study whether tyrothricin differentially affected T and/or B cells, splenocyte cultures were treated with ConA to stimulate T cells and with LPS to stimulate B cells, respectively, either in the presence or the absence of different concentrations (0.1–2 µM) of tyrothricin (Figure 2). Viability of the immune cells was measured by Alamar blue assay. The viability of T cells was impaired in a dose-dependent manner, reaching approximatively 50% reduction upon addition of 1 µM tyrothricin (Figure 2A; *p* < 0.005). However, no impact on the viability of proliferating B cells stimulated with LPS was noted at any of the concentrations used (Figure 2B).

**Figure 3:**
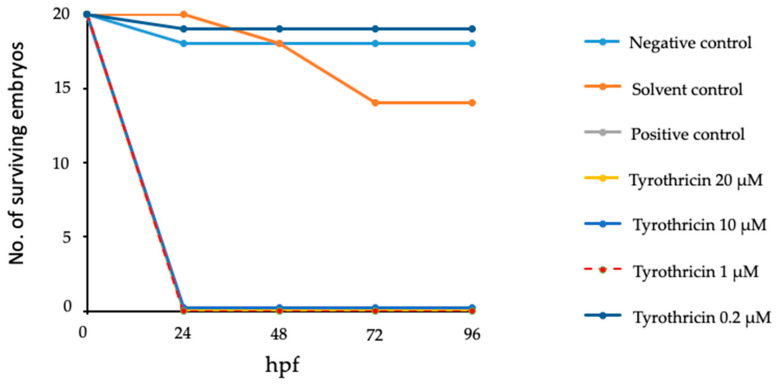
Survival curves for zebrafish embryos upon exposure to E3 medium (Negative control), the solvent DMSO (Solvent control), the positive control drug BKI-1750 (50 µM; Positive control), and Tyrothricin 0.2–20 µM); hpf = hours post fertilization.

Exposure of fertilized zebrafish eggs to 1, 10, and 20 µM tyrothricin (Table 2) led to death of all embryos, similarly to the positive control BKI-1750 (50 µM). Malformations were only seen in the negative control and the solvent controls. As can be seen in Figure 3, mortality of zebrafish embryos upon exposure to 1–20 µM tyrothricin took place within 24 h. In contrast, tyrothricin applied at 0.2 µM did not affect the viability of zebrafish embryos and also did not induce any malformations.

### 3.3. Tyrothricin Treatment of T. gondii Tachyzoites Transiently Induces Structural Alterations

**Figure 4:**
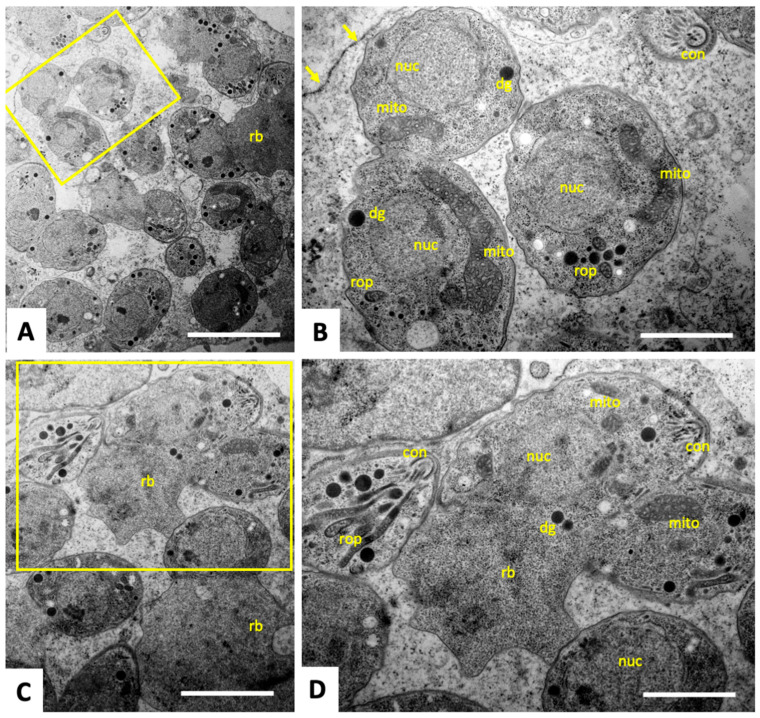
TEM of non-treated *T. gondii* tachyzoites. The yellow boxed area in (**A**,**C**) are shown at higher magnification in (**B**,**D**), respectively; nuc = nucleus, con = conoid, rop = rhoptries, dg = dense granules, mito = mitochondrion; rb = residual body. Arrows point to the parasitophorous vacuole membrane. Bars in (**A**) = 1.8 µm; (**B**) = 0.6 µm; (**C**) = 1.1 µm; (**D**) = 0.6 µm.

Tyrothricin-treated and non-treated *T. gondii* Me49 tachyzoites cultured in HFF were comparatively assessed by TEM. As can be seen in control cultures fixed and processed at 24–48 h after infection (Figure 4), tachyzoites maintained in the absence of compound underwent intracellular proliferation by endodyogeny within a parasitophorous vacuole, separated from the cytoplasm by a parasitophorous vacuole membrane. The typical features of apicomplexan parasites, such as the apical conoid, secretory organelles such as rhoptries, micronemes and dense granules, a single nucleus, and parts of the single mitochondrion with cristae and electron-dense matrix (Figure 4B,D), were clearly discernible. Frequently tachyzoites undergoing proliferation and still attached to the residual body were detected (Figure 4A,C,D).

**Figure 5:**
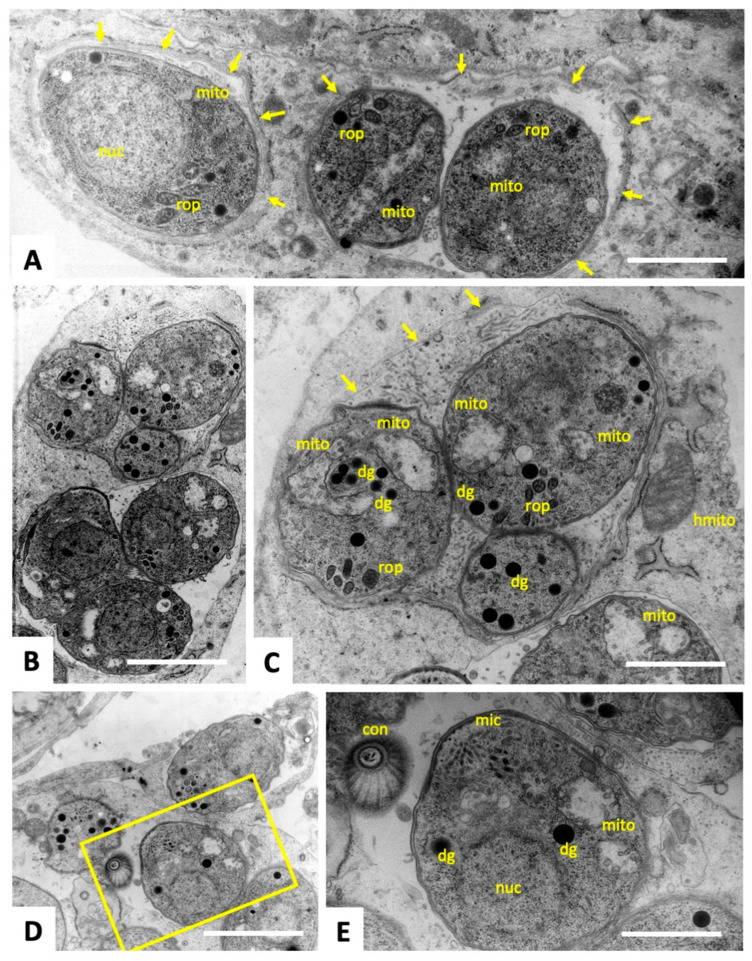
TEM of *T. gondii* tachyzoites treated with 0.5 µM tyrothricin for 6 h (**A**), 24 h (**B**,**C**), and 48 h (**D**,**E**). The yellow boxed area in (**D**) is shown at higher magnification in (**E**); nuc = nucleus, con = conoid, rop = rhoptries, dg = dense granules, mito = mitochondrion; hmito = host cell mitochondrion; Arrows point to the parasitophorous vacuole membrane. Bars in (**A**) = 0.6 µm; (**B**) = 1 µm; (**C**) = 0.5 µm; (**D**) = 1 µm µm; (**E**) = 0.5 µm.

At 6 h after initiation of drug treatments with tyrothricin (Figure 5A), distinct changes in some, but not all, intracellular parasites were detectable, especially concerning the mitochondrion, the matrix of which had lost its distinct electron-dense texture, and cristae appeared to dissolve (Figure 5A). At 24 h (Figure 5B,C) and 48 h (Figure 5D,E), these changes became progressively more pronounced, resulting in tachyzoites with an almost empty mitochondrion or its residues, partially containing debris of matrix and cristae components. The mitochondrial membrane, however, as well as the apical conoid, secretory organelles, the parasitophorous vacuole membrane, and the overall cytoplasmic organization were not profoundly disturbed.

**Figure 6:**
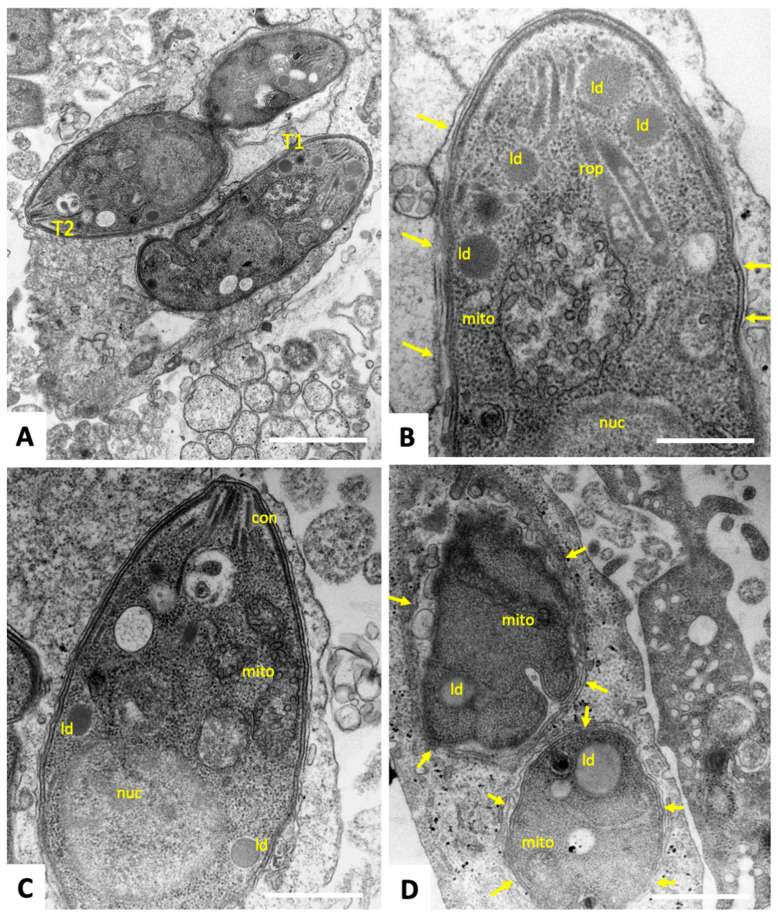
TEM of *T. gondii* tachyzoites grown in HFF and treated with 0.5 µM tyrothricin for 72 (**A**–**C**) and 96 h (**D**). The two tachyzoites labeled T1 and T2 in (**A**) are shown at higher magnification in (**B**,**C**), respectively; mito = mitochondrion, con = conoid, rop = rhoptries; ld = lipid droplets. Arrows point towards the parasitophorous vacuole membrane. Bars in (**A**) = 1 µm; (**B**) = 0.4 µm; (**C**) = 0.5 µm; (**D**) = 1 µm.

Interestingly, at 72–96 h after initiation of treatment (Figure 6), the parasite mitochondrial matrix started to regain a more electron-dense appearance, cristae were more clearly visible again, and larger inclusions filled with amorphous material, most likely lipid droplets, had accumulated in the tachyzoite cytoplasm at both the anterior and posterior part. Thus, the effect of tyrothricin seemed to be of only a transient nature, and parasites exhibited a marked recovery over time.

### 3.4. Tyrothricin Affects the Mitochondrial Membrane Potential (MMP)

**Figure 7:**
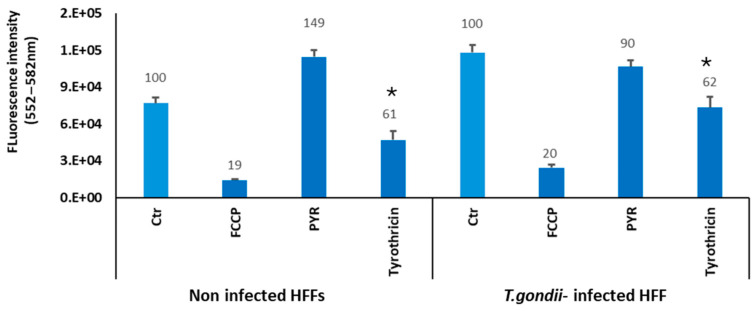
Measurements of the mitochondrial membrane potential by TMRE uptake in non-infected HFF and *T. gondii*-infected HFF. Cells were grown in T25 culture flasks and incubated in the presence or absence of the uncoupler FCCP, pyrimethamine (PYR), and tyrothricin. The bars represent the mean of TMRE fluorescence, with standard deviations calculated from three biological replicates. TMRE uptake of 100% was set for the control cells without drug treatments, and the percentage of TMRE fluorescence intensity in relation to the controls is displayed on the top of each bar. Data comparison between groups was performed using Student’s *t*-test, and * indicates *p* < 0.05.

As TEM showed that tyrothricin initially affected the ultrastructure of the mitochondrial matrix and cristae in *T. gondii* tachyzoites, the potential impact of tyrothricin on the MMP in *T. gondii*-infected HFF and uninfected HFF cultures was investigated by measuring TMRE uptake. The known MMP inhibitor carbonyl cyanide 4-(trifluoromethoxy) phenylhydrazone (FCCP) was used as positive control, decreasing TMRE uptake by 80% compared to untreated cultures (Figure 7). Pyrimethamine (Pyr), the standard anti-*Toxoplasma* drug interfering in folate metabolism, did not impact the MMP in tachyzoites or HFF, thus no change in TMRE uptake was noted. Tyrothricin treatment in uninfected as well as in *T. gondii*-infected HFF cultures resulted in a decrease in TMRE uptake of 40% in both cases, indicating that tyrothricin affects the MMP in both *T. gondii* tachyzoites as well as in HFF.

### 3.5. The Tyrothricin Component Tyrocidine, but Not Gramicidin, Is Active Against T. gondii Tachyzoites and Impairs HFF and Zebrafish Embryo Viability

Tyrothricin is a mixture of *Brevibacillus brevis*-derived polypeptides, and consists of around 60% tyrocidines and 40% gramicidins. Tyrocidines comprise four cyclic decapeptides (tyrocidine A, B, C and D) with distinct amino acids placed on positions 3, 4, and 7. Gramicidins are a mixture of linear ionophore peptides gramicidin A, B, and C with 15 amino acids each forming helices, and the cyclodecapeptide gramicidin S composed of two identical pentapeptides joined head to tail [39]. In order to verify whether the cyclic decapeptide or the linear ionophore peptide affects *T-gondii* proliferation, tyrocidine A and gramicidin A were used. As demonstrated in Table 3, gramicidin A, applied at 1 µM, exhibited no effect on *T. gondii* proliferation or viability of HFF. In contrast, tyrocidine A strongly impacted the proliferation of *T. gondii* tachyzoites but also affected HFF viability at 0.1 and 1 µM (Table 4). Overall, this suggested that tyrocidine A was responsible for mediating the anti-parasitic activity and cytotoxicity of tyrothricin.

Treatments of freshly fertilized zebrafish embryos (Table 4) confirmed these findings, showing that tyrocidine A at concentrations ranging from 1 to 20 µM induced mortality in 20 out of 20 zebrafish embryos, while at 0.2 µM, only 4 out of 20 embryos were lethally damaged. In contrast, gramicidin A did not impair embryo viability at any of the concentrations used, but malformations occurred in 4 out of 20 embryos at the highest concentration (20 µM).

### 3.6. Identification of Tyrocidine A and Gramicidin A Binding Proteins in T. gondii Extracts by DAC-MS-Proteomics

**Figure 8:**
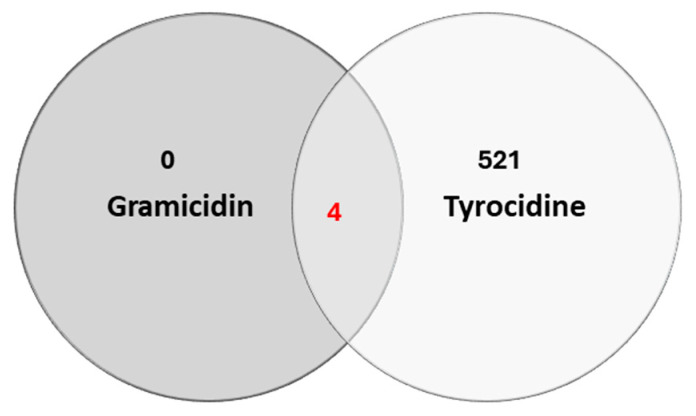
Numbers of tyrocidine- and/or gramicidin-binding proteins in cell-free extracts of *T. gondii.* Venn diagram detailing the distribution of identified proteins (526) which were not binding to the mock column.

To characterize proteins that bind specifically to tyrocidine A, differential affinity chromatography (DAC) was combined with mass spectrometry (MS)-based quantitative proteomics of respective eluates. Gramicidin A coupled to the same matrix was used as a non-active control peptide. Overall, 525 proteins were identified, of which 521 were exclusively bound to tyrocidine A, and four proteins bound to both peptides tyrocidine A and gramicidin A (Figure 8). No proteins binding to gramicidin A or the mock columns were identified. The entire dataset is included in Supplementary Table S2.

The four proteins that bound to both tyrocidine and gramicidin columns are shown in Table 5. They are ranked according to their relative abundance based on IBAQ values. Among these four proteins, the SAG-related sequence SRS20A (TGME49_285870) was by far the most abundant.

Supplementary Table S2 provides a list of the 521 proteins binding specifically to tyrocidine A, and the top 20 with regard to relative abundance are summarized in Table 6. Among these, the dense granule protein GRA1 encoded by TGME49_270250 was the most abundant (rAbu 8.4). Notably, an additional 23 GRA proteins were shown to bind specifically to tyrocidine A (see Supplementary Table S2). The hypothetical protein, which is homologous to the cytochrome c oxidase subunit ApiCOX30 encoded by TGME49_297810, was the second most abundant protein binding to tyrocidine A. In addition, 49 proteins functionally related to mitochondrial activities were identified in the tyrocidine A-binding fraction (see Supplementary Table S2). The third and fourth most abundant proteins were a putative calcium binding protein precursor encoded by TGME49_229480 and the SAG-related sequence SRS52A (Table 6), respectively. A total of 30 SRS proteins were identified as specifically binding to tyrocidine A, four of which were among the 20 most abundant proteins listed in Table 6. Within the group of 20 most abundant proteins, almost half (10/20) were secretory proteins (SRS, dense granule, microneme, or rhoptry proteins), and overall, 80 of the 521 tyrocidine A-binding proteins were annotated as secretory components.

## 4. Discussion

In this study, we aimed to explore the therapeutic potential and mode of action of several AMPs for the treatment of toxoplasmosis. The selected AMPs were chosen based on previous reports of their activity against various protozoan parasites, including *Plasmodium* and *Leishmania*, as well as other pathogenic organisms. Among the tested AMPs, only tyrothricin showed inhibitory activity against *T. gondii*, but also had a small therapeutic window and exhibited cytotoxic effects in host cells and zebrafish embryos. The safety concerns associated with tyrothricin are well known [40], as its broad antimicrobial activity is accompanied by significant systemic toxicity. In fact, the cytotoxicity of AMPs mediated through cationic and hydrophobic components is an inherent property of membrane-active AMPs [41], and is usually more pronounced for broad-spectrum AMPs, as they are more likely to exhibit off-target effects [42,43].

Despite these obvious shortcomings, several studies suggested that distinct AMPs demonstrated promising antiparasitic activity against *T. gondii*, though with varying degrees of cytotoxicity toward host cells. For instance, an alkaline peptide extracted from *Haemaphysalis longicornis* was shown to induce pore formation and limit the proliferation of *T. gondii* tachyzoites [44]. Another peptide, derived from *Conus californicus*, reduced the invasion and proliferation of *T. gondii* in host cells [45], and α-helical cationic peptides extracted from *Helicobacter pylori* reduced the survival rate of *T. gondii* tachyzoites and inhibited adhesion and invasion of macrophages [46]. Lycosin-I, α-helical peptide from spider venom, suppressed *T. gondii* proliferation both in vitro and in vivo, accompanied by altered expression of cytokine transcripts (e.g., reduced IL-6/IL-8) in infected HFF [15]. Another spider-derived polypeptide, XYP1, and its derivatives were reported to suppress invasion and proliferation of *T. gondii* and to extend the survival time of infected mice, most likely by dampening the host inflammatory reaction [47,48]. An AMP derived from the broad-spectrum AMP leucinostatin exhibited promising in vitro efficacy against *T. gondii* tachyzoites and limited host cell cytotoxicity but induced adverse side effects in the mouse model. In that particular study, DAC-MS-proteomics suggested that these adverse effects could be due to multiple interactions with host immune cell components, resulting in higher cerebral parasite loads in treated mice compared to untreated infected mice [18].

TEM analysis revealed early mitochondrial alterations and multilayered membrane formation around the parasitophorous vacuole in tyrothricin-treated *T. gondii* tachyzoites. The origin of the additional membranes surrounding the vacuole remains unclear, and it is not known whether they are produced by *T. gondii* itself or are derived from the host cell. Coppens and Joiner showed that host cell cholesterol is incorporated into the parasitophorous vacuole membrane during invasion [49]. Importantly, the presence of multilamellar membrane structures surrounding the PV following tyrothricin exposure mirrors findings by Romano et al. [50], who showed that host-derived autophagic membranes can be recruited to the PV under conditions of host cell stress or nutrient deprivation. This suggests that the membrane accumulations observed in the present study may originate from the host cell, possibly via autophagic or endosomal pathways hijacked by the parasite. The absence of known *T. gondii* pathways capable of de novo lipid bilayer synthesis further supports a host-derived origin for these membranes [51]. Similar ultrastructural changes at the parasitophorous vacuole membrane are consistent with recently reported host–parasite membrane interactions and stress responses induced by thiosemicarbazone drugs [52]. This additional membrane material may influence nutrient acquisition by the parasite and could potentially contribute to the observed mitochondrial disruptions and cytoplasmic vacuolization of tachyzoites.

Mitochondrial swelling and dissolution of the mitochondrial matrix and cristae structures in *T. gondii* have been documented upon treatments with drugs that target specifically the mitochondrion, such as decoquinate [53], but also upon treatments with other compounds such as trithiolato-bridged arene ruthenium complexes [54], the leucinostatin derivative 6027 [18], or particularly with agents that disrupt lipid homeostasis or membrane integrity, such as the ionophoric agent monensin [55]. Membrane perturbation is a common feature among antimicrobials with disruptive effects on ion gradients and membrane dynamics. Likewise, ref. [56] reported cytoplasmic vacuolization and mitochondrial dysfunction in parasites treated with artemisinin derivatives, further supporting the idea that mitochondrial alterations represent a generalized stress response in *T. gondii*. As shown here, treatment with tyrothricin at 0.5 µM for 3.5 h resulted in a significant (~40%) reduction in TMRE fluorescence in both infected and uninfected cultures, suggesting a moderate but consistent depolarization of the mitochondrial membrane in both host and parasite cells, with more dramatic effects on the parasites, since tachyzoites, in contrast to HFF, contain only one mitochondrion. The moderate depolarization observed upon tyrothricin treatment, though less severe than that induced by the positive control FCCP, indicates that the mitochondrial function is partially impaired, and this is in agreement with ultrastructural observations.

It is important to note that both effects, namely the formation of the multi-layered parasitophorous vacuole membrane, as well as the lesions within the mitochondrial matrix, become less evident after 72 and 96 h of treatment with tyrothricin, suggesting that the effect is transient, and at least partially reversible, even in the presence of the compound. This points to the inherent adaptive potential of *T. gondii* tachyzoites and possibly their host cells, documented also for other compounds [52,57].

The fact that tyrothricin not only displays considerable anti-*T. gondii* activity but also impairs the viability of HFF and immune cell subsets as well as zebrafish embryos renders this compound unsuitable for further in vivo evaluation, as the risk of adverse effects in animal studies was considered to be high. However, we decided to investigate which proteins could be actually targeted by this AMP. Tyrothricin is composed of tyrocidines and gramicidins. We here show, by using two members of each peptide group, tyrocidine A and gramicidin A, that in the case of *T. gondii*, the cyclic peptide tyrocidine A is the active compound, while the linear peptide gramicidin A is ineffective. Similar findings were obtained in *Plasmodium*, which was affected by tyrocidines, but not notably by gramicidin S [58,59]. In Gram-positive bacteria such as *Listeria monocytogenes*, tyrocidine A exhibits ionophoric properties and can disrupt membrane permeability [60,61], and forms defined ion-conducting pores, induces lipid phase separation, and strongly reduces membrane fluidity, resulting in delocalization of a broad range of peripheral and integral membrane proteins [62]. Tyrocidine A was also shown to be active against the pathogenic fungus *Aspergillus fumigatus* [63] and *P. falciparum* [59]. Interestingly, tyrocidine A also causes DNA damage and interferes with DNA-binding proteins, indicating that other mechanisms of action besides membrane perturbations could be involved [61].

To identify potential tyrocidine A targets, or interaction partners, *T. gondii* extracts were analyzed by DAC and mass spectrometry, revealing 521 proteins specifically binding to tyrocidine A. A similarly large number of potential interaction partners (269 proteins) was found by *T. gondii*-DAC-MS proteomics using the leucinostatin derivative AMP-6027 [18]. However, in that study, binding of AMP-6027 was assessed in a *T. gondii* RH strain, which is a type I strain. Only four proteins were found to bind to both tyrocidine A and gramicidin A. No protein was identified to bind only to gramicidin A, which coincides with the absence of anti-parasitic activity. On the other hand, it appears conceivable that an AMP capable of interacting with multiple proteins would exert higher anti-parasitic activity, as well as cytotoxicity. Most likely not all tyrocidine A-binding proteins identified here are actual drug targets, since the circular cationic peptide is prone to bind to anionic parasite proteins. Whether these are relevant in terms of target interactions is not clear, and remains to be investigated.

Of the 521 proteins identified in the fraction binding only to tyrocidine A, the dense granule (GRA) protein TgGRA1 (TGME49_270250) was found to be the most abundant. Similarly to other GRA proteins, TgGRA1 is secreted into the parasitophorous vacuole and plays an important role in acquiring nutrients from the host cell and enabling other GRA proteins to reach the parasitophorous vacuole membrane. Disrupting GRA1 function leads to metabolic defects as well as structurally altered mitochondria, with an overall inhibition in proliferation [64]. The observed interaction suggests that TgGRA1 may represent a molecular target for tyrocidine A anti-parasitic activity.

The second-most abundant tyrocidine A-binding protein was a hypothetical protein encoded by TGME49_297810, which is a homolog of the apicomplexan-specific cytochrome c oxidase subunit ApiCOX30. ApiCOX30 is part of the mitochondrial respiratory complex IV and plays a critical role in oxidative phosphorylation in *T. gondii* [65]. The identification of eight additional components of the mitochondrial respiratory chain in the tyrocidine A-binding fraction is consistent with the structural impairment of the mitochondrial matrix and cristae observed by TEM, and the disruption of the mitochondrial membrane potential, implicating direct interference with mitochondrial electron transport as a potential antiparasitic mechanism. Another mitochondrial protein, but associated with the outer mitochondrial membrane and ranked sixth in relative abundance, is the eukaryotic porin protein (TGME49_263300). This protein is a voltage-gated anion channel, involved in the import of proteins, metabolites, and other small molecules into the mitochondrion [66]. Deletion of TGME49_263300 leads to impairment of *T. gondii* proliferation and severe structural and functional alterations in the mitochondrion and the endoplasmic reticulum, thus similar features as seen upon treatments with tyrothricin. Of note, a homolog of TGME49_263300 named TGRH88_06771 was identified in high abundance in *T. gondii* RH tachyzoites as AMP-6027 binding protein [18].

The tyrocidine A-binding fraction also included a considerable number of *T. gondii* tachyzoite surface proteins. Overall, 30 SAG-related sequence SRS proteins were identified as specifically binding to tyrocidine, four of which were found in the 20 proteins with the highest relative abundance. SRS proteins constitute the major surface antigen family in *T. gondii* and are central to host cell attachment, immune modulation, and stage-specific surface expression [67,68,69]. The preferential binding of tyrocidine A to these proteins suggests that it may interact directly with the parasite surface, potentially disrupting critical processes required for infection. Particularly in Gram-positive bacteria, tyrothricin is known for its membrane-disrupting properties, integrating into lipid bilayers to form ion-permeable channels [70]. This interaction with membrane-associated proteins in *T. gondii*, such as SRS antigens, raises the possibility that tyrothricin could similarly impair parasite membrane stability or interfere with surface protein functionality.

In addition to SRS proteins, secretory microneme or rhoptry proteins implicated in host cell interaction and/or invasion were identified in the 20 most abundant proteins of the tyrocidine A-binding fraction. Among those are TgPDI (TGME49_211680), which is implicated in the interaction with basigin, a well-known receptor on the surface of astrocytes mediating *T. gondii* infection [71], as well as the microneme protein M2AP (TGME49_214940), which is associated with the microneme protein TgMIC2, a major virulence factor [72]. Additional microneme proteins are TgMIC11 (TGME49_204530), functionally implicated in the perforin-like protein 1 (PLP1)-mediated egress of *T. gondii*, facilitating membrane disruption and parasite dissemination [73], and TgAMA1 (TGME49_255260), a protein that mediates *T. gondii* attachment to host cells but is not essential for host cell invasion [74]. Besides the two rhoptry proteins toxofilin (TGME49_214080) and ROP6 (TGME49_258660) found among the 20 most abundant proteins, an additional 11 rhoptry proteins were identified, among them TgRON2, which interacts with TgAMA1 during the formation of the moving junction as the parasite enters the host cell [75].

In addition, a putative calcium-binding protein precursor (TGME49_229480), also known as TgpCaBP, was among the prominent tyrocidine-binding proteins, with the third-highest ranking. TgpCaBP comprises four EF-hand motifs responsible for Ca^2+^-binding and is localized in the cytosol and ER of *T. gondii* tachyzoites. TgpCaBP-deficient parasites exhibit impaired motility, lower rates of host cell invasion, proliferation, and egress [76]. In addition, three members of the small calcium-binding calmodulin-like proteins were identified among the tyrocidine binding fraction (TGME49_305050, TGME49_269442, and TGME49_249240), which upon activation interact with various targets including motor proteins, ion channels, kinases, phosphatases, and membrane transport proteins [77,78], playing a major role in host cell invasion [79]. Additional members of the tyrocidine A-binding fraction are calcium-dependent protein kinases such as TgCDPK1 (TGME49_301440) and TgCDPK3 (TGME49_305860), implicated in signaling events leading to host cell invasion and egress through, e.g., regulation of microneme secretion [80,81]. We have not specifically addressed the question of whether tyrocidine affects the host cell invasion process, but of note is the fact that the IC50 is slightly lower when the drug is added concomitantly to infection as opposed to when the parasites are already intracellular. Thus, tyrothricin could dysregulate signaling pathways relevant in host cell entry.

## 5. Conclusions

We here showed that tyrothricin, a complex mixture of AMPs, exhibits profound anti-*Toxoplasma* activity, and that the activity is largely based on the linear peptide tyrocidine A. By using DAC-MS-proteomics, a large number of *T. gondii* proteins were found to bind to tyrocidine A, indicating that multiple targets could be involved in the mode of action.

The fraction of *T. gondii* proteins binding to the previously investigated leucinostatin derivative AMP-6027 was dominated by the Sec61β family protein (TGRH88_008910), which alone accounted for 9% of all identified binders, followed by GRA12 and a eukaryotic porin protein. The prominence of Sec61β, a component of the ER translocon, pointed toward a potential interaction of leucinostatins with protein trafficking or translocation machinery, in addition to organellar targets. In contrast, tyrocidine A (1) associates with dense granule and surface antigen proteins, potentially affecting host–parasite interface dynamics; (2) targets mitochondrial components such as the electron transfer chain at the inner, and porins at the outer, mitochondrial membrane, consistent with observed mitochondrial dysfunction; and (3) could interfere with calcium-regulated signaling pathways essential for parasite replication and egress. This contrast underscores how peptide structure (cyclic versus linear) can shape protein–ligand interactions and may contribute to distinct intracellular mechanisms of action against *T. gondii*. Together, these results point to a multimodal mechanism of tyrocidine A action in *T. gondii*.

## Figures and Tables

**Table 1:** Primary screening of AMPs on the in vitro proliferation of *T. gondii* β-gal tachyzoites and the viability of HFF, respectively. Viability was measured by Alamar blue assay, and proliferation was assessed by β-galactosidase assay. * Values indicate percentages of proliferation and viability ± standard deviation in relation to controls without compounds (% of control ± SD).

	HFF		*T. gondii* β-Gal	
	* Viability		* Proliferation	
AMP	0.1 μM	1 μM	0.1 μM	1 μM
Cecropin	153 ± 13	62 ± 20	113 ± 11	98 ± 5
Phylloseptin-1	123 ± 3	60 ± 17	105 ± 16	101 ± 10
Melitin	167 ± 16	64 ± 15	133 ± 6	104 ± 14
Temporin B	125 ± 12	114 ± 2	108 ± 14	106 ± 10
Tyrothricin	83 ± 12	62 ± 10	49 ± 5	3 ± 2
LL-37	111 ± 15	129 ± 17	95 ± 11	89 ± 3
BMAP-18	111 ± 16	96 ± 11	107 ± 6	103 ± 7

Viability and proliferation percentages of experimental compounds were calculated relative to controls by multiplying these values by 100 and dividing by the control values.

**Table 2:** Acute embryo toxicity of tyrothricin in zebrafish embryos. Freshly fertilized zebrafish eggs were exposed to four different drug concentrations (0.2–20 μM). Malformations and mortality were assessed every 24 h until 96 h post-fertilization.

	Neg. Control	Solvent Control	Pos. Control BKI-1750	Tyrothricin			
Conc. (µM)			50	20	10	1	0.2
Mortality	2/20	3/20	20/20	20/20	20/20	20/20	1/20
Malformations	4/20	3/20	0/20	0/20	0/20	0/20	0/20
Non-affected	14/20	14/20	0/20	0/20	0/20	0/20	19/20

**Table 3:** Impact of tyrothricin components tyrocidine A and gramicidin A on the viability of uninfected HFF and the proliferation of *T. gondii*-β-gal tachyzoites cultured in HFF. AMPs were assessed at 0.1 and 1 µM. Values indicate percentages of proliferation of *T. gondii* tachyzoites and viability of HFF ± standard deviations in relation to controls without compounds (% of control ± SD).

	HFF		*T. gondii* β-Gal	
	Viability		Proliferation	
	0.1 μM	1 μM	0.1 μM	1 μM
Gramicidin A	108 ± 19	115 ± 0	107 ± 19	118 ± 8
Tyrocidine A	30 ± 9	22 ± 8	81 ± 20	9 ± 5

**Table 4:** Acute toxicity assessment of tyrocidine A and gramicidin A in zebrafish embryos. Fertilized zebrafish eggs were exposed to four different drug concentrations (0.2–20 μM), and mortality/malformations were recorded in a blinded manner until 96 h post-fertilization.

	Negative Control	Solvent Control	Tyrocidine A				Gramicidin A			
Conc. (µM)			20	10	1	0.2	20	10	1	0.2
Mortality	2/20	3/20	20/20	20/20	20/20	4/20	0/20	0/20	0/20	0/20
Malformations	4/20	3/20	0/20	0/20	0/20	1/20	4/20	0/20	0/20	0/20
Non-affected	14/20	14/20	0/20	0/20	0/20	15/20	16/20	20/20	20/20	20/20

**Table 5:** List of the four proteins binding to both tyrocidine A and gramicidin A columns as identified by DAC followed by mass spectrometry. See Supplementary Table S2 for the full dataset. The relative abundances (rAbu) are based on iBAQ sum up to a total of 1,000,000 for each sample. The proteins are listed according to their decreasing rAbu values.

ToxoDB ORF	Annotation	rAbu
TGME49_285870	SAG-related sequence SRS20A	89
TGME49_221620	beta-tubulin, putative	8
TGME49_250770	eukaryotic initiation factor-4A, putative	3
TGME49_261740	hypothetical protein	1

**Table 6:** List of the 20 most abundant proteins specifically binding to tyrocidine A as identified by DAC followed by mass spectrometry. See Supplementary Table S2 for the full dataset. The relative abundances (rAbu) are based on iBAQ sum up to a total of 1,000,000 for each sample. The proteins are listed according to their decreasing rAbu values.

ToxoDB ORF	Annotation	rAbu
TGME49_270250	dense granule protein GRA1	8.4
TGME49_297810	hypothetical protein (cytochrome c oxidase subunit ApiCOX30)	3.8
TGME49_229480	calcium binding protein precursor, putative	3.3
TGME49_315320	SAG-related sequence SRS52A	3.3
TGME49_233480	SAG-related sequence SRS29C	2.7
TGME49_263300	voltage-dependent anion-selective channel protein	2.3
TGME49_308840	SAG-related sequence SRS51	1.9
TGME49_211680	protein disulfide isomerase	1.5
TGME49_268850	enolase 2	1.4
TGME49_214940	MIC2-associated protein M2AP	1.3
TGME49_308020	SAG-related sequence SRS57	1.3
TGME49_232350	lactate dehydrogenase LDH1	1.2
TGME49_214080	Toxofilin	1.2
TGME49_204530	microneme protein MIC11	1.0
TGME49_236040	fructose-1,6-bisphosphate aldolase	1.0
TGME49_208370	dense granule protein GRA46	1.0
TGME49_258660	rhoptry protein ROP6	0.9
TGME49_221510	cytochrome c oxidase subunit ApiCOX18	0.9
TGME49_224900	adenylate kinase, putative	0.9
TGME49_255260	apical membrane antigen AMA1	0.9

## Data Availability

Data are contained within the article and Supplementary Materials.

## Supplementary Materials

The following supporting information can be downloaded at: https://www.mdpi.com/article/10.3390/biomedicines14010172/s1, Table S1: Panel of antimicrobial peptides used in the first screening of *T. gondii*; Table S2: Identification of tyrocidine A and gramicidin A binding proteins in *T. gondii*: full dataset.
