# Supplementary material for: Potential Molecular Targets of the Broad-Range Antimicrobial Peptide Tyrothricin in the Apicomplexan Parasite Toxoplasma gondii
Source: Biomedicines. 2026 Jan 13;14(1):172. doi: 10.3390/biomedicines14010172 (PMC12838698; doi:10.3390/biomedicines14010172)
Supplement: Supplementary file 1 [file biomedicines-14-00172-s001.zip › biomedicines-4077766-supplementary/Suppl. Table S1.pdf]

Table S1: Structural and Molecular Characterization of AMPs Tested Against Parasitic Organisms

AMP	Sequence	Molecular weight (g/mol)	Parasite	Antiparasitic effect	References
Cecropin CA(1-7) M(2-9)	KWKLFKKIGAVLKVL	1771.32	*L. donovani* /*L. pifanoi*	IC50<1 µM	(Pulido et al., 2012) [25].
Phylloseptin-1	FLSLIPHAINAVSAIAKHN	2016.39	*L. amazonensis*	IC50 = 0.5 µM	(Pulido et al., 2012) [25].
Melitin	GIGAVLTTGLPALISWIKRKRQQ	2507.03	Epimastigote Trypomastigote Amastigote	IC50 =1.4 µg.mL^{-1}	(El-Dirany et al., 2021) [27]
Temporin B	LLPIVGNLLKSLL	1392.80	L. pifanoi amastigotes	LC50=8.6 µM	(El-Dirany et al., 2021) [27]
Tyrothricin	cyclo [VOLFPFFNQY]	1228	*P. falciparum* Parasite Stage **:** Erythrocytic stages	IC50 = 0.6 nM	(Pulido et al., 2012) [25].
LL-37	LLGDFFRKSKEKIGKEFKRIVQRIKDFLRNLVPRTES	4493.34	L. donovani promastigotes and amastigotes<br><br>L. major amastigotes	IC_{50} = 20 µM	(El-Dirany et al., 2021) [27]
BMAP-18	GRFKRFRKKFKKLFKKLS	2342.96	Trypanosoma	25 mg/mL	(Haines et al., 2009) [22].

Pulido, D.; Rivas, L.; Torrent, M.; Andreu, D. Antimicrobial Peptide Action on Parasites. *Curr. Drug Targets* **2012**, *13*, 1138–1147. https://doi.org/10.2174/138945012802002393

El-Dirany, R.; Shahrour, H.; Dirany, Z.; Abdel-Sater, F.; Gonzalez-Gaitano, G.; Brandenburg, K.; Martinez de Tejada, G.; Nguewa, P.A. Activity of Anti-Microbial Peptides (AMPs) against *Leishmania* and Other Parasites: An Overview. *Biomolecules* **2021,** *11,* 984. https://doi.org/10.3390/biom11070984.

Haines, L.R.; Thomas, J.M.; Jackson, A.M.; Eyford, B.A.; Razavi, M.; Watson, C.N.; Gowen, B.; Hancock, R.E.W.; Pearson, T.W. Killing of Trypanosomatid Parasites by a Modified Bovine Host Defense Peptide, BMAP-18. *PLoS Negl. Trop. Dis.* **2009**, *3*, e373. https://doi.org/10.1371/JOURNAL.PNTD.0000373.
